# A Hybrid CNN-SVM Approach for ECG-Based Multi-Class Differential Diagnosis of PTSD, Depression, and Panic Attack

**DOI:** 10.3390/bios16010052

**Published:** 2026-01-10

**Authors:** Parisa Ebrahimpour Moghaddam Tasouj, Gökhan Soysal, Osman Eroğul, Sinan Yetkin

**Affiliations:** 1 Biomedical Device Technology, Vocational School of Health Services, Ankara Medipol University, Ankara 06050, Türkiye; 2 Department of Electrical and Electronics Engineering, Ankara University, Ankara 06830, Türkiye; 3 Department of Biomedical Engineering, TOBB University of Economics and Technology, Ankara 06510, Türkiye; erogul@etu.edu.tr; 4 Department of Psychiatry, Ankara Gülhane Health Application and Research Hospital, University of Health Sciences, Ankara 06010, Türkiye; sinan.yetkin@sbu.edu.tr

**Keywords:** depression, differential diagnosis, electrocardiogram, hybrid deep learning, panic attack, post-traumatic stress disorder

## Abstract

Background: PTSD diagnosis is challenging. Symptoms overlap with depression and panic attacks. This causes misdiagnosis and delayed treatment. Current methods lack objective biomarkers. This study presents a hybrid AI framework. It combines CNNs and SVMs. The system detects PTSD from ECG signals. Methods: ECG data from 79 participants were analyzed. Four groups were included. PTSD patients numbered 20. Depression patients numbered 20. Panic attack patients numbered 19. Healthy controls numbered 20. Wavelet transform created scalograms. Three CNN models were tested. AlexNet, GoogLeNet, and ResNet50 were used. Deep features were extracted. SVMs classified the features. Five-fold validation was performed. Statistical tests confirmed significance. Results: Hybrid models performed robustly. ResNet50 + SVM and AlexNet + SVM achieved statistically equivalent results with accuracies of 97.05% and 97.26%, respectively. AUC reached 1.00 for multi-class tasks. PTSD detection was highly accurate. The system distinguished PTSD from other disorders. Hybrid models beat standalone CNNs. SVM integration improved results significantly. Conclusions: This is the first ECG-based AI for PTSD diagnosis. The hybrid approach achieves clinical-level accuracy. PTSD is distinguished from depression and panic attacks. Objective biomarkers support psychiatric assessment. Early intervention becomes possible.

## 1. Introduction

Post-traumatic stress disorder (PTSD) represents one of the most debilitating psychiatric conditions, characterized by intrusive re-experiencing, avoidance behaviors, negative alterations in cognition and mood, and hyperarousal symptoms [1]. Recent studies show that about 3.6% of adults in the U.S. experience PTSD each year, while lifetime prevalence is estimated at 6.8% in the U.S. and 3.9% across the world population [2]. Women are disproportionately affected by PTSD, with lifetime prevalence nearly twice as high in women (10–12%) as in men (5–6%). A similar disparity is evident in adolescence, where prevalence is 8.0% for females compared to 2.3% for males [3]. PTSD imposes a substantial economic burden, with annual costs reaching 232.2 billion in the United States [4], while European data demonstrates healthcare expenditures three times higher than controls, with lifetime costs approximating €43,000 per patient [5].

The clinical differentiation of PTSD presents significant diagnostic challenges due to substantial symptom overlap with panic attacks and major depressive disorder [6]. Post-traumatic stress disorder (PTSD) and panic attacks are pretty similar in terms of autonomic symptoms; panic attacks are reported as a secondary symptom in approximately 30–60% of PTSD patients [7]. The diagnostic complexity is further amplified by PTSD’s substantial comorbidity with major depressive disorder, as approximately 52% of individuals with PTSD meet criteria for comorbid depression, while 36–61% of patients presenting with primary depression harbor undiagnosed PTSD [8,9,10]. The diagnostic confusion stems from shared symptom clusters including anhedonia, emotional numbing, sleep disturbances, concentration difficulties, and social withdrawal [11]. Patients with dual PTSD-depression diagnoses exhibit reduced treatment response rates and longer recovery trajectories [12]. This diagnostic overlap necessitates sophisticated approaches to differential diagnosis, as misclassification can lead to suboptimal treatment selection [13].

The diagnostic confusion stems from convergent effects on shared cardiovascular pathways, particularly through dysregulation of the hypothalamic-pituitary-adrenal (HPA) axis and sympathoadrenal system [14]. Meta-analytic evidence demonstrates that PTSD confers a 55–61% increased risk of coronary heart disease [15], with all three disorders triggering excessive catecholamine release, resulting in identical acute cardiovascular manifestations including elevated heart rate, blood pressure fluctuations, and altered heart rate variability [16]. While anxiety states activate both HPA and sympathoadrenal axes simultaneously, panic attacks demonstrate predominant sympathetic activation with minimal HPA involvement [17]. The chronic dysregulation leads to sustained cardiovascular risk through endothelial dysfunction, accelerated atherosclerosis, increased inflammatory marker expression, and altered autonomic nervous system balance [18]. Recent research utilizing the Trier Social Stress Test reveals that PTSD patients exhibit blunted acute stress responses with slower cardiovascular recovery and reduced heart rate variability [19]. These shared pathophysiological mechanisms underscore the need for objective, signal-based diagnostic approaches [20].

The emergence of deep learning (DL) technologies has revolutionized psychiatric disorder classification by providing unprecedented capabilities to extract complex patterns from neuroimaging and physiological signals [21]. Recent studies have demonstrated the effectiveness of deep learning techniques in detecting cardiovascular diseases, identifying ECG arrhythmias, and performing automated health classification [22,23,24,25,26].

Recent bibliometric analyses reveal rapid growth in DL applications for mental health disorders, with over 2811 research publications demonstrating CNN accuracies exceeding 98% for depression, schizophrenia, and anxiety disorders [27,28]. Neuroimaging-based DL approaches utilizing EEG, fMRI, and structural MRI have demonstrated remarkable success, with CNN-LSTM hybrid architectures showing superior performance in capturing both spatial and temporal features [29,30]. Multimodal deep learning algorithms analyzing EEG, fNIRS, and neuroimaging data yielded significant results, achieving 97.26% classification accuracy for schizophrenia detection and 94.34% for generalized anxiety disorder [31,32]. However, current DL approaches face significant limitations, including small heterogeneous datasets, lack of external validation, and the inability to effectively differentiate between disorders with overlapping symptomologies such as PTSD, depression, and panic attacks [33]. This technological gap highlights the urgent need for innovative DL methodologies [34].

Despite remarkable achievements in psychiatric disorder classification through neuroimaging, ECG-based DL for PTSD detection remains largely unexplored [35]. While existing research has successfully utilized DL for cardiac pathology detection, psychiatric applications remain predominantly confined to binary PTSD versus control classification [36,37]. CWT emerges as the optimal solution, generating scalogram representations that simultaneously preserve time localization and frequency decomposition, creating rich visual patterns that CNNs can effectively process [38]. The scalogram representation maintains non-stationary characteristics of cardiac signals, captures transient events crucial for psychiatric state identification, and provides multi-resolution analysis across different time scales [39]. Recent methodological advances demonstrate that CWT-based scalograms enable CNNs to achieve superior performance in cardiac signal classification tasks [40]. However, current ECG-based psychiatric classification research faces significant limitations, including a focus on binary classification and the absence of comprehensive frameworks for distinguishing between overlapping psychiatric conditions [41].

Despite recent advances in artificial intelligence applications for mental health diagnostics, prior studies have predominantly focused on binary classification tasks (e.g., PTSD vs. control) and have not fully leveraged ECG-based features for multi-class differentiation. In addition, no comprehensive framework currently integrates time–frequency representations with hybrid deep and machine learning architectures to address overlapping psychiatric conditions.

To address these critical research gaps, this study introduces the first comprehensive framework for ECG-based multi-class psychiatric disorder classification through two key innovations. First, we develop a deep learning system capable of simultaneously differentiating PTSD, depression, panic attacks, and healthy control states from ECG signals, advancing beyond existing binary classification approaches to clinically relevant differential diagnosis. Second, we propose a novel hybrid CNN-SVM architecture that combines ResNet50’s automatic feature extraction with SVM’s robust classification performance, enhanced through PCA for optimal dimensionality reduction. This hybrid approach transforms CWT-derived scalograms into discriminative features that outperform individual CNN or traditional machine learning methods. Our framework employs rigorous 5-fold cross-validation and explores multiple ECG segment lengths to optimize diagnostic accuracy.

The main contributions of this study are summarised as follows:

- A novel multi-class ECG-based diagnostic framework is introduced for differentiating PTSD, major depression, panic disorder, and healthy controls, addressing a gap left by prior binary classification studies.
- Time–frequency representations (CWT-based scalograms) are used to capture patterns of autonomic dysregulation relevant to psychiatric conditions.
- Multiple deep learning architectures (AlexNet, GoogLeNet, ResNet50) are systematically compared for the multi-class psychiatric ECG classification task.
- A hybrid CNN–SVM pipeline enhanced by PCA is proposed to combine automatic deep feature extraction with robust machine-learning discrimination.
- Four ECG segment durations (5 s, 10 s, 15 s, 20 s) are evaluated to investigate the effect of temporal resolution on diagnostic accuracy.
- A comprehensive evaluation is conducted using accuracy, precision, recall, F1-score, AUC, and confusion-matrix analyses to identify the best classifier and window length.

## 2. Materials and Methods

**Figure 1:**
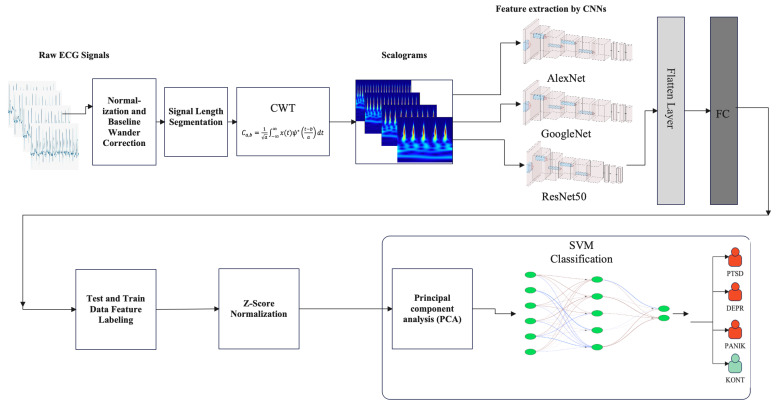
CNNs-SVM framework. (Group abbreviations: PTSD = Post-Traumatic Stress Disorder, DEPR = Depression, PANIK = Panic Disorder, KONT = Healthy Control).

This dual-stage framework leverages direct CNN classification and CNN-based feature extraction combined with SVM classification to maximize diagnostic accuracy. Figure 1 depicts the framework, which consists of: first, raw ECG signals undergo normalization and baseline wander correction to eliminate low-frequency artifacts. Subsequently, the signals are segmented into fixed lengths suitable for time-frequency analysis. In the next stage, the CWT is applied to each segment to generate scalogram images that encode both temporal and spectral characteristics. These scalograms are fed into pre-trained convolutional neural networks (AlexNet, GoogLeNet, and ResNet50) for direct multi-class classification. In parallel, the CNN architectures are employed as feature extractors within a hybrid classification approach. Extracted features are standardized using Z-score normalization and reduced via Principal Component Analysis (PCA) before classification with a Support Vector Machine (SVM).

### 2.1. Dataset and Participants

ECG recordings were obtained from Gülhane Education and Research Hospital (2017–2022) under ethics approval (2024/25). All participants were psychiatrically evaluated and classified into PTSD (n = 20), depression (n = 20), panic attack (n = 19), and healthy control (n = 20) groups.

All ECG recordings were reviewed by a cardiologist to verify technical signal quality (baseline stability, adequate signal-to-noise ratio, minimal artifacts). All 79 recordings met quality standards. This technical validation was independent of psychiatric diagnosis confirmation by psychiatrists. Data were sampled at 200 Hz with electrode impedance 10 kΩ. The dataset is based on 5-min ECG recordings. Each recording contains approximately 60,000 samples. The signals were divided into segments of 5, 10, 15, and 20 s to generate scalogram images. This segmentation process resulted in a significant increase in the total number of images. The number of samples and scalograms for each segment duration are provided in Table 1. For clarity, throughout the manuscript and figures, group labels are abbreviated as follows: PTSD = Post-Traumatic Stress Disorder, DEPR = Major Depression, PANIK = Panic Disorder, and KONT = Healthy Control.

**Figure 2:**
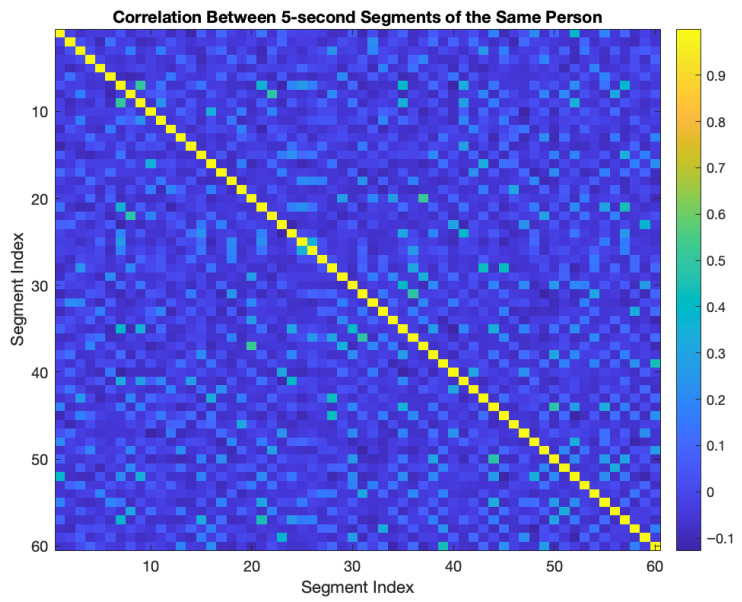
Cross-segment correlation analysis between 5-s segments of the same person ECG signal.

ECG signals are non-stationary signals, with high correlation between consecutive 5-s segments taken from the same person, not expected. 5-s intersegment correlation was calculated from a representative subject. As shown in Figure 2, the 60 × 60 correlation matrix shows that the average correlation is very low (−0.0038, std: 0.0943) and the segments can be evaluated independently.

### 2.2. Preprocessing

#### 2.2.1. Artifact Removal

Baseline Wander represents a low-frequency interference component in ECG recordings that originates from external factors, physiological influences, and environmental noise sources. To address this issue, a finite impulse response (FIR) based high-pass zero-phase filter was implemented with a cutoff frequency set at 0.5 Hz. This filtering approach effectively eliminated low-frequency noise components from the acquired signals. Subsequently, the filtered data underwent Z-score standardization for amplitude normalization. Z-score standardization serves as a crucial preprocessing technique that harmonizes signal magnitudes across different recordings. This normalization strategy facilitates the analysis of heterogeneous signals within a consistent analytical framework by eliminating inter-signal amplitude discrepancies. The implementation of this preprocessing pipeline ensures signal homogeneity and enhances the reliability of subsequent analytical procedures.

#### 2.2.2. Continuous Wavelet Transform (CWT)

CWT helps us to use time-frequency features together by converting one-dimensional ECG signals into two-dimensional scalogram images. CWT effectively extracts non-stationary signal features by providing simultaneous time-frequency analysis of the signal. The resulting scalogram analyzes both low-frequency components (P and T waves) and high-frequency features (QRS complexes) by visualizing temporal frequency changes. CWT is [42]. Mathematically defined as follows:

(1) $$C_{a,b}=\frac{1}{\sqrt{a}}\int_{-\infty }^{\infty }x\left(t\right)\psi^{*}\left(\frac{t-b}{a}\right)dt$$

where *a* represents the scale parameter and *b* denotes the translation parameter. Various wavelet functions, including Bump, Morse, and Morlet wavelets, were evaluated for signal transformation. The Morlet wavelet demonstrated superior performance and was selected for this study due to its optimal frequency resolution characteristics. This transformation preserves both temporal and spectral information, making it particularly suitable for analyzing the dynamic characteristics of ECG signals.

### 2.3. CNN Training and Optimization

#### 2.3.1. Stochastic Gradient Descent (SGD) Optimization

Stochastic Gradient Descent (SGD) is a widely used optimization algorithm in deep learning models. Unlike traditional gradient descent algorithms, SGD calculates gradient values using randomly selected mini-batch samples instead of the entire dataset in each iteration. This method significantly reduces computational cost for large datasets and lowers the risk of getting stuck in local minima due to its stochastic nature. SGD’s basic update rule allows movement in the direction of the cost function gradient in the parameter space. Momentum-enhanced SGD (SGDM) uses weighted averages of previous updates to reduce oscillations in the optimization process and provide more reliable convergence.In this study, the momentum coefficient β = 0.9 and learning rate α = 0.0001 was set:

(2) $$\begin{array}{cc} v_{t} & =\beta v_{t-1}+\alpha \nabla_{\theta }J\left(\theta \right) \end{array}$$

(3) $$\begin{array}{cc} \theta & =\theta -v_{t} \end{array}$$

where $v_{t}$ is the momentum vector, β is the momentum coefficient (e.g., 0.9), α is the learning rate (e.g., 0.0001), $\nabla_{\theta }J\left(\theta \right)$ is the gradient of the cost function, and θ is the parameter being updated [43].

#### 2.3.2. Hyperparameters

In binary and multi-class classification, CNNs were carefully selected with hyperparameters during the training process to ensure optimum performance. A mini-batch size of 20 samples was used to balance computational efficiency with gradient estimation accuracy; this maintained stable convergence while providing sufficient stochastic noise to prevent overfitting.The maximum epoch limit was set to 8 iterations over the entire dataset to prevent excessive memorization of training patterns and ensure good generalization capability. Stochastic Gradient Descent with Momentum (SGDM) was selected as the optimization algorithm due to its ability to accelerate convergence and reduce oscillations in the loss landscape through the incorporation of previous gradient information. The learning rate was conservatively set to 0.0001 to ensure stable weight updates without overshooting optimal solutions, particularly important given the sensitivity of deep networks to parameter changes. Validation frequency was configured to evaluate model performance every 10 training steps, enabling early detection of overfitting and providing regular monitoring of generalization performance throughout the training process. Hyperparameters were selected empirically based on validation performance in preliminary experiments. Hyperparameters were tuned using 20-s segments with AlexNet, then applied to all other configurations without further tuning. Table 2 presents the hyperparameters employed during model training.

All experiments were conducted on a MacBook (Apple Inc., Cupertino, CA, USA) Pro (2019) with Intel Core i5 processor (1.4 GHz), 8 GB RAM, and Intel Iris Plus Graphics 645, using MATLAB R2023a. Table 3 summarizes the training and inference performance across all architectures and segment lengths. Training times decreased substantially with longer segments due to reduced sample counts, while all models achieved real-time inference capability (<0.5 s per 5-min ECG) on consumer-grade hardware without requiring GPU acceleration.

#### 2.3.3. Cross-Validation

Cross-validation is a technique used to evaluate a model’s generalization performance by dividing the dataset into k equal parts. In each iteration, one part is reserved as the test set while the remaining k − 1 parts are used for training. This process is repeated k times to ensure each part is tested. The final accuracy is calculated as the average of all k fold accuracies, providing a more reliable estimate than a single train-test split [44]. The selection of k value is based on the bias-variance trade-off, and selecting the optimal k value by testing different values is a standard practice. In this study, k = 5 was selected for cross-validation.

Cross-validation helps evaluate how well a model performs. The dataset is divided into k equal parts. Each time, one part becomes the test set. The other k − 1 parts are for training. This repeats k times so every part gets tested once. We calculate the final accuracy by averaging all k results. This gives better estimates than splitting data only once. Choosing k depends on balancing bias and variance. Testing different k values is common practice. We used k = 5 in our study.

To prevent data leakage, cross-validation was performed at the participant level. In each fold, all segments from a given participant were assigned exclusively to either the training or the test set, ensuring no participant’s data appeared in both sets within any fold.

### 2.4. Principal Component Analysis (PCA)

Principal Component Analysis (PCA) is a statistical technique used for dimensionality reduction of high-dimensional data. PCA creates a hierarchical coordinate system by finding directions that capture the maximum variance in the data. The algorithm first obtains mean-centred data by subtracting the mean of the data matrix, then performs eigenvalue decomposition of the covariance matrix.

In this study, PCA was applied to high-dimensional feature vectors extracted from CNN fully connected layers, retaining 95% cumulative explained variance to ensure minimal information loss while reducing computational complexity and overfitting risk. For AlexNet, GoogLeNet, and ResNet50 models, dimensionality reduction rates of 72%, 66%, and 61% were achieved, respectively. The PCA is mathematically defined as:

(4) $$\begin{array}{cc} \bar{X} & =\frac{1}{n}\sum_{i=1}^{n}X_{i} \end{array}$$

(5) $$\begin{array}{cc} B & =X-\bar{X} \end{array}$$

(6) $$\begin{array}{c} C=\frac{1}{n-1}B^{T}B \end{array}$$

(7) $$\begin{array}{c} CV=VD \end{array}$$

(8) $$\begin{array}{cc} T & =B\times V \end{array}$$

where $\bar{X}$ is the mean of the data matrix *X*, *B* is the mean-centered data matrix, *C* is the covariance matrix, *D* is the diagonal matrix of eigenvalues, *V* contains the corresponding eigenvectors obtained from the eigendecomposition of *C*, and *T* represents the transformed data in the principal component space [45]. Table 4 presents the original feature dimensions and the reduced dimensions after PCA for various CNN models and segment lengths.

### 2.5. Evaluation Metrics for Multi-Class Classification

In multi-class classification problems, performance evaluation employs micro and macro averaging approaches, which offer different computational methods and perspectives. The micro averaging approach aggregates the TP, TN, FP, and FN values across all classes to compute a single metric. In this approach, class sizes (sample counts) influence the results, with larger classes carrying greater weight. The macro averaging approach, conversely, calculates metrics separately for each class and then takes the arithmetic mean of these metrics. Grandini et al. [46] provided a comprehensive examination of multi-class classification metrics. This method is independent of class sizes and provides a balanced evaluation by assigning equal weight to each class.

## 3. Results

### 3.1. Multi-Class Classification Performance

Three different CNN architectures were evaluated for PTSD, depression, panic attacks, and multi-class classification of healthy control groups at varying segment lengths. The AlexNet model achieved an overall accuracy of 94.85% with an MCC value of 0.93 (Table 5). The GoogLeNet model showed improved performance, yielding an accuracy of 96.14% and an MCC of 0.95 (Table 6). The ResNet50 model achieved the highest performance on 5-s segments, with an overall accuracy of 96.65%, an MCC value of 0.96, and a micro-AUC of 0.998 (Table 7). In PTSD classification, ResNet50 provided the best metrics, achieving an accuracy of 95.70%, a sensitivity of 94.67%, and a false positive rate of 4.30%.

Three CNN architectures were tested. These classified individuals with PTSD, depression, panic attacks, and healthy individuals. Different segment lengths were tried. ResNet50 yielded the best results. This model worked with 5-s segments. Overall accuracy was 96.65%. The MCC value was 0.96, and the micro-AUC value was 0.998. GoogLeNet showed different results. This model had an accuracy of 96.14% and an MCC value of 0.95. AlexNet performed worse, with an accuracy of 94.85% and an MCC value of 0.93. In PTSD classification, ResNet50 stood out. Accuracy was 95.70%, sensitivity was 94.67%, and the false positive rate was 4.30%. Clinically significant FOR values remained low in all models, ranging from 1.49% to 1.94%. This indicates a very low risk of missing PTSD cases. In all models, MCC values were above 0.92, indicating the strong correlation required for a psychiatric diagnosis. All models yielded the best results in 5-s segments. Performance decreased as segment duration increased. This finding is consistent with the acute cardiovascular features of PTSD attacks and supports the clinical significance of short ECG analysis windows.

### 3.2. Hybrid CNNs-SVM Multi-Class Classification

To further strengthen classification robustness, features extracted by CNN architectures were integrated with SVM classifiers following PCA dimensionality reduction. This hybrid approach combines the deep feature learning capacity of convolutional neural networks with the discriminative power of support vector machines, demonstrating superior performance in multi-class psychiatric disorder discrimination tasks.

SVM Configuration: Multi-class classification employed Error-Correcting Output Codes (ECOC) with linear kernel binary learners (MATLAB fitcecoc). Linear kernel was selected for its computational efficiency and appropriateness for PCA-transformed feature space. Features were standardized using Z-score normalization before PCA dimensionality reduction (95% variance retention), then classified via SVM.

The hybrid models consistently improved performance compared to CNN-only configurations across all segment lengths, with particularly pronounced advantages for shorter temporal segments. Notably, 5-s segments showed the most distinct class separations in PCA space, with each psychiatric condition forming well-defined clusters. As segment length increased to 20 s, class boundaries became increasingly diffuse, highlighting the critical impact of temporal resolution on feature extraction quality from ECG scalograms.

**Figure 3:**
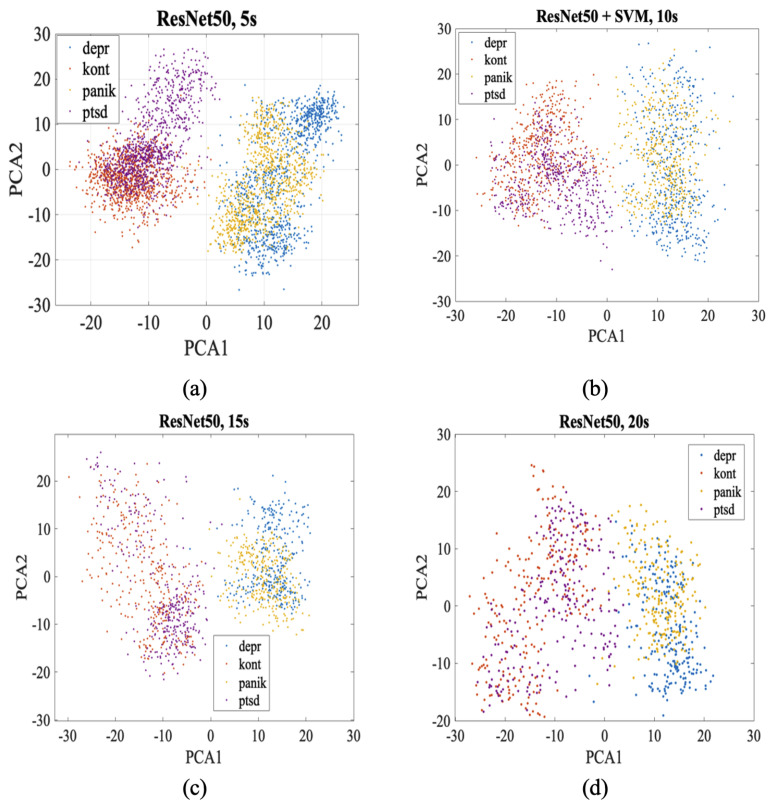
ResNet50 + SVM hybrid model PCA visualization across different segment lengths ((**a**) 5 s, (**b**) 10 s, (**c**) 15 s, (**d**) 20 s).

Figure 3 illustrates the PCA visualization of ResNet50 features across different ECG segment lengths, revealing the critical impact of temporal resolution on classification performance. In 5-s segments (a), the four psychiatric classes form remarkably distinct clusters in PCA space, with depression (blue) creating a compact cluster in the lower right, control group (red) concentrated in the lower left, panic attack (yellow) positioned in the middle right, and PTSD (purple) clearly separated in the upper left region. As segment length increases to 10 s (b), class separations remain preserved but cluster boundaries begin to soften slightly. At 15 s (c), inter-class distinctions show more pronounced degradation, particularly between control and depression groups, while PTSD maintains its characteristic position. In 20-s segments (d), inter-class overlaps reach maximum levels with all groups exhibiting more diffuse distributions, though general class tendencies are still preserved. This progressive degradation emphasizes that cardiovascular manifestations of psychiatric disorders require short-duration, high-resolution analysis for optimal discrimination.

For AlexNet + SVM, the best performance was obtained with 5 s segments, achieving 97.26% overall accuracy, 0.96 MCC, and a micro-AUC of 1.00 (Table 8). PTSD classification reached 95.95% precision and 96.91% recall, with a low FDR of 4.04%, highlighting the model’s ability to minimize false negatives. Performance gradually decreased with longer durations, with accuracy dropping to 90.97% and MCC to 0.88 at 20 s.

Similarly, GoogleNet + SVM achieved its peak performance at 5 s with 96.35% accuracy, 0.95 MCC, and 0.99 micro-AUC (Table 9). Precision and recall for PTSD were 94.20% and 94.83%, respectively. Despite robust results, a noticeable decline was observed for 15 s and 20 s, where accuracy fell to 91.33% and 90.31%, confirming the importance of short-segment ECG windows.

The ResNet50 + SVM hybrid achieved the best overall performance. With 5 s segments, it reached 97.05% accuracy, 0.97 MCC, and a nearly perfect micro-AUC of 1.00 (Table 10). PTSD classification yielded 95.98% precision and 95.50% recall, with the lowest FDR (1.29%) among all models. Although performance slightly declined at longer durations (overall accuracy 91.48% at 20 s), the hybrid ResNet50 maintained consistently higher MCC values compared to AlexNet and GoogleNet.

**Figure 4:**
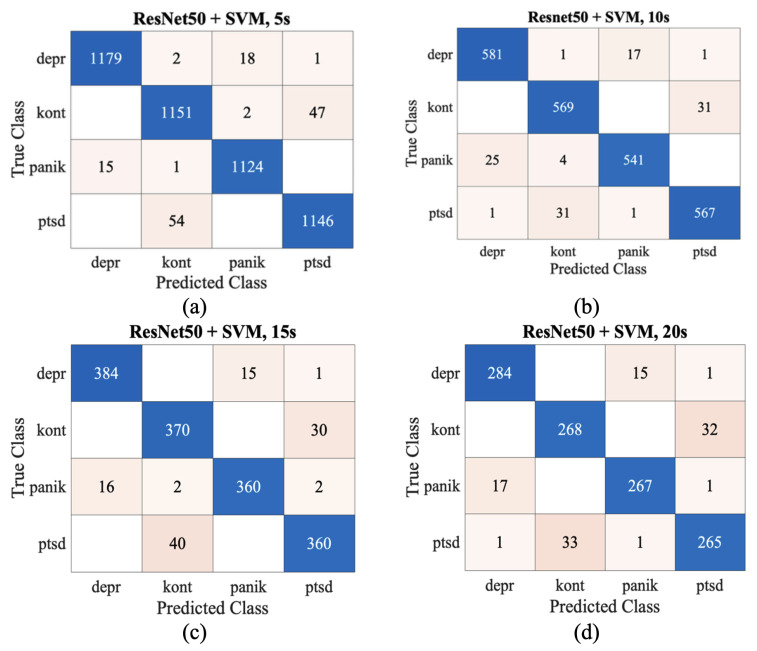
ResNet50 + SVM hybrid model confusion matrices across different segment lengths ((**a**) 5 s, (**b**) 10 s, (**c**) 15 s, (**d**) 20 s).

**Figure 5:**
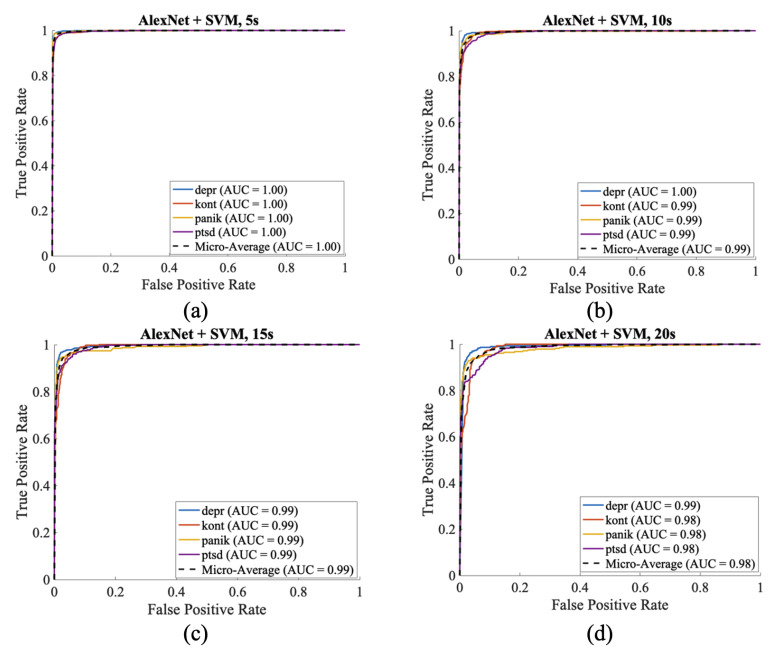
ResNet50 + SVM hybrid model ROC curves and AUC values across different segment lengths. ((**a**) 5 s, (**b**) 10 s, (**c**) 15 s, (**d**) 20 s).

The confusion matrices in Figure 4 illustrate that ResNet50 + SVM provided the clearest class separation with 5 s segments, while misclassifications increased at 10–20 s. Corresponding ROC curves in Figure 5 confirmed near-perfect separability at 5 s, with only minor reductions at longer durations. In summary, the CNNs-SVM hybrids demonstrated superior stability and generalization over CNN-only models. Among them, ResNet50 + SVM emerged as the most reliable, combining strong precision–recall balance with minimal false discovery rates. These findings highlight the potential of hybrid frameworks as clinically meaningful diagnostic tools for psychiatric ECG analysis, especially in minimizing the risk of missing PTSD cases.

### 3.3. Statistical Analysis

#### 3.3.1. Statistical Significance Analysis of Resnet + SVM vs. CNN Models

The statistical significance of the observed performance differences was evaluated in Table 11. Paired *t*-tests and Wilcoxon tests were applied to the 5-fold cross-validation results. When the effect of SVM integration was examined, a 0.49% improvement (*p* = 0.009) for ResNet50 and a 2.41% improvement (*p* = 0.007) for AlexNet were found to be statistically significant, while no significant improvement was observed for GoogLeNet (*p* = 0.548). In comparing the hybrid models, AlexNet + SVM achieved the highest accuracy (97.26%) and performed marginally better than ResNet50 + SVM (97.05%) (*p* = 0.037). The difference between ResNet50 + SVM and GoogLeNet + SVM was not statistically significant (*p* = 0.086). These results confirm that SVM integration provides real performance gains, especially for ResNet50 and AlexNet architectures.

#### 3.3.2. McNemar’s Test for Prediction-Level Comparison on Hybrid CNN + SVM Models

To complement fold-level paired tests, we performed McNemar’s tests comparing prediction-level disagreements between models. Table 12 presents the results.

McNemar’s test revealed no significant difference between ResNet50 + SVM and AlexNet + SVM (*p* = 0.502), indicating statistically equivalent performance despite the 0.21% mean accuracy difference. Both models significantly outperformed GoogLeNet + SVM (*p* < 0.05).

### 3.4. Error Analysis and Misclassification Patterns

Error analysis was performed on hybrid CNN + SVM models with 5-s segments. These results are shown in Table 13. PTSD-Control confusion was most pronounced, while PTSD-Depression and PTSD-Panic confusions were minimal. AlexNet + SVM and Resnet50 + SVM showed the best overall discrimination. This confirms the specificity of ECG biomarkers in differentiating PTSD from other psychiatric disorders. Based on these results, PTSD is confused with the control group, and depression and panic attacks are also confused with each other.

### 3.5. Performance of Traditional Machine Learning Approaches

Table 14 presents the performance of conventional machine learning approaches using handcrafted statistical features extracted from ECG signals. These features include amplitude-based parameters (peak value, RMS, mean), variation measures (standard deviation, skewness, kurtosis), signal geometry descriptors (shape factor, crest factor, clearance factor, impulse factor), and signal quality metrics (SNR, SINAD, THD). All features were computed in MATLAB and selected based on their established effectiveness in biomedical signal processing for distinguishing pathological and healthy ECG patterns [35].

Traditional machine learning approaches achieved overall accuracies ranging from 32–44%, barely exceeding chance performance (25% for 4-class classification). The best-performing traditional method, Linear SVM, achieved 44.30% accuracy, while the worst-performing method, Neural Network, achieved only 31.65%.

**Figure 6:**
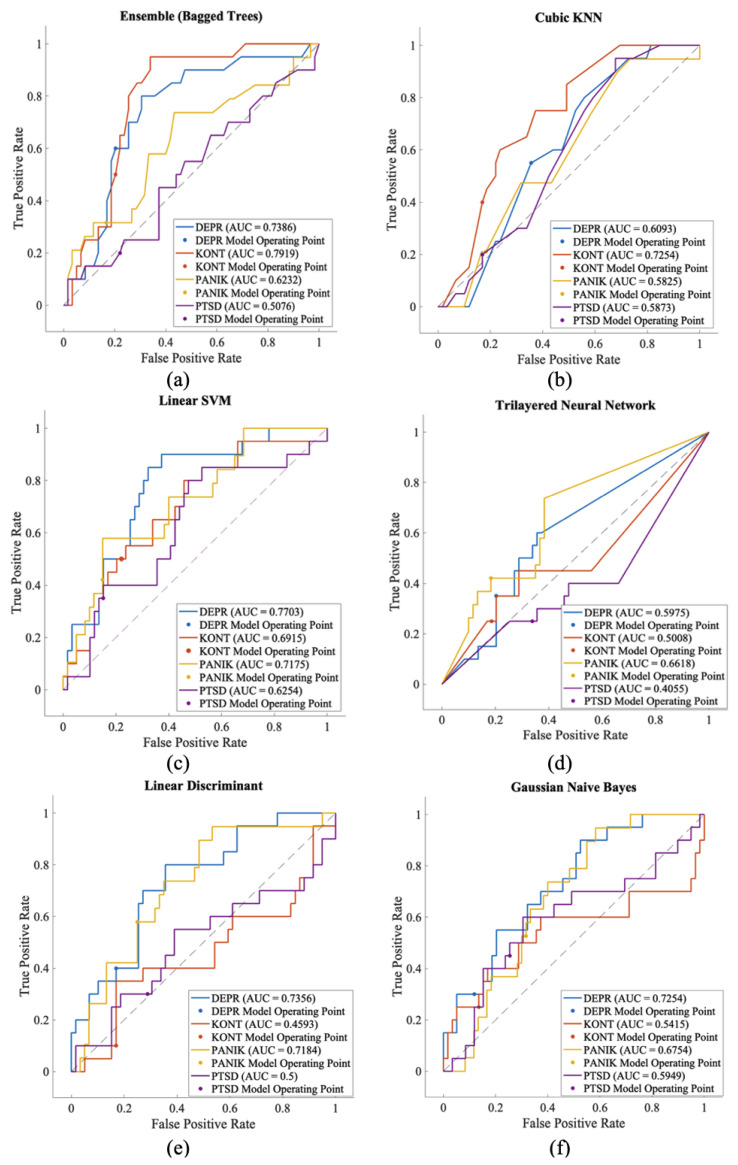
ROC analysis examines the class-based performance of machine learning models in the multiple classification problem.

The ROC analysis in Figure 6 clearly demonstrates the performance differences between classes. AUC values range from 0.405 to 0.791. The highest performance was obtained for the control class in the ensemble model with an AUC of 0.791. The lowest performance was observed for the PTSD class in the Three-Layer Neural Network with an AUC of 0.405. This wide performance range reflects the complexity of multiple classifications. These results demonstrate the necessity of deep learning for this complex multi-class psychiatric classification task, as our proposed hybrid CNN-SVM approach achieves approximately twice the accuracy compared to conventional methods.

## 4. Discussion

This study is based on ECG recordings from 79 participants in a single center. Collecting psychiatric ECG datasets is challenging, even for retrospective data, due to ethical constraints, expert requirements, and multicenter validation processes. Despite this limitation, the model demonstrated stable performance in layered cross-validation. To address potential data leakage concerns, we employed strict subject-wise cross-validation ensuring no participant’s segments appeared in both training and validation sets. However, our study lacks an independent test set, representing an inherent trade-off given our limited sample size. The reported performance reflects internal validation rather than true external generalization. Future studies will focus on multicenter external validation to establish clinical generalizability and assess model robustness across diverse populations.

Hyperparameters were tuned on one configuration (20 s-AlexNet) and applied to others. Nested cross-validation would provide more conservative estimates, but was computationally prohibitive. However, our best performance came from an untested configuration (5 s-ResNet50: 97.05% vs. 20 s-AlexNet: 91.48%), mitigating concerns about overfitting.

The second important aspect is longitudinal ECG monitoring. Repeated recordings or wearable measuring devices make it possible to assess temporal symptom dynamics and treatment response. This addresses the limitation of the single-session 5-min recordings used in this study. However, our approach partially compensates for this deficiency by dividing the 5-min ECG into shorter segments. These shorter windows capture more subtle autonomous changes and allow CNN models to extract deeper time-frequency features.

Explainability remains an open problem in deep learning. Current attribution methods do not provide fully reliable physiological interpretations for clinical decision-making. Future studies will integrate SHAP-based feature attribution and waveform-level analyses to better understand the cardiac dynamics driving model decisions. Recent studies have explored multimodal physiological fusion combining ECG with phonocardiogram (PCG), electrodermal activity (EDA), respiration, or accelerometry. The reference BSPC study [47] demonstrates that integrating complementary modalities increases feature diversity. However, these approaches require multiple synchronous physiological signals that are not routinely accessible in standard psychiatric clinics. Our study deliberately focused on a single-modality ECG framework to assess whether autonomous signatures alone carry sufficient discriminatory information for multiclass psychiatric discrimination. This design is consistent with the practical limitations of real-world psychiatric assessment, where ECG is often the only routine physiological signal collected. However, multimodal fusion represents a valuable future direction, particularly for capturing complementary autonomous and behavioral markers.

In terms of computational feasibility, the model was trained and tested on a standard CPU-based workstation (Intel Core i5, 8 GB RAM) without GPU acceleration. Despite the modest hardware, inference per scalogram required only tens of milliseconds, demonstrating suitability for real-time or near-real-time deployment in future devices. This system is not designed as a standalone diagnostic tool, but rather as a decision support tool that can complement existing scales such as PCL-5, HAM-D, and clinical interviews.

## 5. Conclusions

This study presents, for the first time, a hybrid CNN-SVM approach based on ECG signals for differentiating PTSD, depression, and panic attacks. The method yielded diagnostic accuracy exceeding 97%. ResNet50+SVM and AlexNet+SVM models showed similar performance. Scalogram representations enabled simultaneous time-frequency analysis. CNN architectures extracted complex physiological features that couıd be found manually. Five-second ECG segments were found to be optimal for capturing abrupt cardiovascular changes associated with psychiatric symptoms. Multiclass classification successfully differentiated PTSD from depression and panic attacks. Error analysis showed minimal confusion between PTSD and other psychiatric disorders. PTSD-Control interference was the main error pattern, confirming that these ECG biomarkers are objective diagnostic indicators. The hybrid CNN-SVM framework goes beyond existing binary classification approaches and offers a clinically ready diagnostic support system. This study lays a methodological foundation for future psychiatric disorder detection using cardiovascular biomarkers, enabling early intervention and reducing misdiagnosis rates.

## 6. Future Work

Future research will focus on enhancing the interpretability of the proposed hybrid CNN–SVM framework through explainable artificial intelligence (XAI) methods such as SHAP (SHapley Additive exPlanations) analysis. This will allow the identification of the most discriminative ECG-derived features contributing to PTSD, depression, and panic attack classification, providing physiological insight into disorder-specific cardiac dynamics. Additionally, extending the dataset to include larger and more diverse populations, as well as testing model generalizability across multi-center ECG databases, will further validate its clinical applicability. Integrating real-time analysis modules into portable ECG monitoring systems may also facilitate early detection and continuous psychiatric assessment in clinical settings. Longitudinal ECG datasets will also be incorporated in future research to enable monitoring of symptom evolution and clinical recovery trajectories. Future studies may also examine multimodal fusion (e.g., ECG + EDA or ECG + respiration), particularly in settings where additional physiological channels can be acquired reliably. Finally, future studies will explore nested cross-validation frameworks with separate inner and outer loops to enable more robust hyperparameter optimization and unbiased performance estimation, particularly in larger and more diverse datasets.

## Figures and Tables

**Table 1:** Scalogram counts by segment duration.

Dur.	Samples	Scal./Rec.	Records	Total
5 s	1000	60	79	4740
10 s	2000	30	79	2370
15 s	3000	20	79	1580
20 s	4000	15	79	1185

**Table 2:** Summary of hyperparameters used in neural network training.

Hyperparameter	Value	Description
Mini-batch size	20	Balances gradient stability with computational efficiency.
Maximum epochs	8	Prevents overfitting and excessive memorization of training patterns.
Optimizer	SGDM	Provides stable convergence by incorporating momentum into gradient updates.
Learning rate	0.0001	Ensures stable and controlled weight updates for sensitive deep networks.
Momentum	0.9 (default)	Accelerates SGD updates and reduces oscillations.
Validation frequency	10 iterations	Enables frequent monitoring of generalization performance.

**Table 3:** Computational Performance Summary.

Hardware: MacBook Pro (2019), Intel Core i5 1.4 GHz, 8 GB RAM, MATLAB R2023a							
**Training Performance (minutes per fold)**							
**Model**	**Type**	**Params (M)**	**5 s**	**10 s**	**15 s**	**20 s**	**Total (5 s)**
AlexNet	CNN only	60.9	31	22	16	11	2.6 h
AlexNet + SVM	Hybrid *	60.9 + SVM	31	22	16	11	2.6 h
GoogLeNet	CNN only	6.9	51	46	29	21	4.3 h
GoogLeNet + SVM	Hybrid *	6.9 + SVM	51	46	29	21	4.3 h
ResNet50	CNN only	25.5	269	121	71	49	22.4 h
ResNet50 + SVM	Hybrid *	25.5 + SVM	269	121	71	49	22.4 h
**Model**	**Type**	**Per Segment**		**Per 5-min ECG**		**Real-time**	
AlexNet	CNN only	11–13 ms		∼0.7 s (60 seg)		Yes	
AlexNet + SVM	Hybrid	11–13 ms		∼0.7 s (60 seg)		Yes	
GoogLeNet	CNN only	18–21 ms		∼1.2 s (60 seg)		Yes	
GoogLeNet + SVM	Hybrid	18–21 ms		∼1.2 s (60 seg)		Yes	
ResNet50	CNN only	24–28 ms		∼1.5 s (60 seg)		Yes	
ResNet50 + SVM	Hybrid	25–29 ms		∼1.6 s (60 seg)		Yes	
**Clinical Deployment Feasibility**							
Training: One-time cost of 2.6–22.4 h (5 s segments); pre-trained models deployable							
Inference: Real-time capable (<2 s per 5-min ECG); CPU-only sufficient							
Hardware: Consumer-grade laptop; no GPU required; model size: 98–240 MB							

* Includes SVM training (<1 min), negligible compared to CNN time. 5 s segments used in this study (optimal accuracy); longer segments reduce training time. SVM classification adds ∼1 ms overhead per segment (negligible). 60 segments per 5-min ECG (300 s ÷ 5 s = 60 segments).

**Table 4:** Feature dimensions before and after PCA for CNN models.

Model	FC Feature Dimension		PCA Reduced Dimension	
	Training Set	Test Set	Training Set	Test Set
AlexNet_5s	3792 × 4096	948 × 4096	3792 × 1149	948 × 1149
AlexNet_10s	1896 × 4096	474 × 4096	1896 × 738	474 × 738
AlexNet_15s	1264 × 4096	316 × 4096	1264 × 518	316 × 518
AlexNet_20s	948 × 4096	237 × 4096	948 × 436	237 × 436
GoogleNet_5s	3792 × 1024	948 × 1024	3792 × 352	948 × 352
GoogleNet_10s	1896 × 1024	474 × 1024	1896 × 306	474 × 306
GoogleNet_15s	1264 × 1024	316 × 1024	1264 × 273	316 × 273
GoogleNet_20s	948 × 1024	237 × 1024	948 × 238	237 × 238
Resnet50_5s	3792 × 2048	948 × 2048	3792 × 802	948 × 802
Resnet50_10s	1896 × 2048	474 × 2048	1896 × 638	474 × 638
Resnet50_15s	1264 × 2048	316 × 2048	1264 × 531	316 × 531
Resnet50_20s	948 × 2048	237 × 2048	948 × 448	237 × 448

**Table 5:** AlexNet Multi-class Classification Performance Across Different Segment Lengths.

Segment Length	Class	Prec. (%)	Recall (%)	Spec. (%)	F1 (%)	FDR (%)	FOR (%)	MCC	AUC	Class Acc. (%)	Overall Acc. (%)	Overall MCC	Micro-AUC
5 s	depr	96.20	92.91	98.75	94.53	3.80	2.37	0.927	0.994	92.91	**94.85**	**0.93**	0.994
	kontrol	95.03	95.66	98.30	95.34	4.96	1.47	0.938	0.994	95.66			
	panik	98.81	95.26	97.66	94.02	7.17	1.51	0.921	0.993	95.26			
	ptsd	92.82	95.58	98.41	95.46	4.65	1.49	0.939	0.995	95.58			
10 s	depr	94.33	93.86	97.96	94.10	5.67	2.22	0.920	0.992	93.86	91.75	0.89	**0.998**
	kontrol	89.33	91.62	96.19	90.46	10.67	2.93	0.871	0.987	91.62			
	panik	91.44	92.42	97.78	91.93	8.57	1.94	0.898	0.989	92.42			
	ptsd	91.83	89.30	97.03	90.55	8.17	3.96	0.871	0.985	89.30			
15 s	depr	84.98	94.75	94.32	89.60	15.02	1.85	0.860	0.985	94.75	89.43	0.86	0.967
	kontrol	87.41	93.75	95.42	90.47	12.59	2.17	0.872	0.974	93.75			
	panik	93.96	81.84	98.33	87.48	6.04	5.52	0.842	0.967	81.84			
	ptsd	**93.05**	87.00	97.80	89.92	6.95	4.31	0.867	0.954	87.00			
20 s	depr	88.29	93.00	95.82	90.58	11.71	2.42	0.873	0.984	93.00	88.52	0.85	0.970
	kontrol	86.32	88.33	95.25	87.31	13.68	3.99	0.830	0.963	88.33			
	panik	91.82	86.67	97.56	89.17	8.18	4.15	0.859	0.973	86.67			
	ptsd	88.06	86.00	96.05	87.02	11.95	4.71	0.827	0.958	86.00			

**Table 6:** GoogleNet Multi-class Classification Performance Across Different Segment Lengths.

Segment Length	Class	Prec. (%)	Recall (%)	Spec. (%)	F1 (%)	FDR (%)	FOR (%)	MCC	AUC	Class Acc. (%)	Overall Acc. (%)	Overall MCC	Micro-AUC
5 s	depr	97.22	98.92	99.04	98.06	2.78	0.37	0.974	0.998	98.92	**96.14**	**0.95**	**0.996**
	kontrol	94.01	95.42	97.94	94.71	5.99	1.56	0.929	0.995	95.42			
	panik	98.65	95.97	99.58	97.29	1.35	1.27	0.965	0.997	95.97			
	ptsd	94.88	94.25	98.28	94.57	5.12	1.94	0.927	0.995	94.25			
10 s	depr	86.62	98.17	94.86	92.03	13.38	0.65	0.894	0.992	98.17	91.22	0.88	0.985
	kontrol	91.09	92.00	96.95	91.54	8.91	2.72	0.887	0.990	92.00			
	panik	97.76	84.04	99.39	90.38	2.24	4.84	0.880	0.979	84.04			
	ptsd	91.25	90.33	97.06	90.79	8.75	3.27	0.877	0.987	90.33			
15 s	depr	90.00	94.50	96.44	92.20	9.79	1.90	0.897	0.988	94.50	90.82	0.88	0.982
	kontrol	87.71	92.75	95.59	90.16	12.29	2.49	0.868	0.982	92.75			
	panik	93.61	88.68	98.08	91.08	6.39	3.50	0.884	0.984	88.68			
	ptsd	92.57	87.25	97.63	89.83	7.43	4.21	0.866	0.975	87.25			
20 s	depr	87.82	91.33	95.71	89.54	12.18	2.98	0.859	0.986	91.33	86.84	0.82	0.977
	kontrol	85.86	83.00	95.37	84.41	14.14	5.70	0.793	0.971	83.00			
	panik	91.11	86.32	97.33	88.65	8.89	4.26	0.852	0.977	86.32			
	ptsd	83.07	86.67	94.01	84.83	16.93	4.59	0.796	0.973	86.67			

**Table 7:** ResNet50 Multi-class Classification Performance Across Different Segment Lengths.

Segment Length	Class	Prec. (%)	Recall (%)	Spec. (%)	F1 (%)	FDR (%)	FOR (%)	MCC	AUC	Class Acc. (%)	Overall Acc. (%)	Overall MCC	Micro-AUC
5 s	depr	98.58	98.00	99.52	98.29	1.42	0.68	0.977	0.998	98.00	**96.65**	**0.96**	**0.998**
	kontrol	94.42	95.83	98.08	95.12	5.58	1.42	0.935	0.996	95.83			
	panik	97.99	98.16	99.36	98.07	2.01	0.58	0.975	0.999	98.16			
	ptsd	95.70	94.67	98.56	95.18	4.30	1.80	0.936	0.996	94.67			
10 s	depr	94.75	96.17	98.19	95.45	5.25	1.31	0.939	0.995	96.17	94.77	0.93	0.994
	kontrol	92.90	96.00	97.51	94.42	7.10	1.80	0.923	0.993	96.00			
	panik	96.33	95.33	98.83	95.83	3.67	1.58	0.939	0.994	95.33			
	ptsd	94.58	92.00	98.30	93.27	5.42	2.71	0.911	0.992	92.00			
15 s	depr	94.00	95.00	98.10	94.50	6.00	1.50	0.933	0.993	95.00	92.50	0.90	0.991
	kontrol	89.60	92.00	96.00	90.78	10.40	2.50	0.890	0.989	92.00			
	panik	95.67	92.67	98.67	94.14	4.33	2.33	0.926	0.992	92.67			
	ptsd	90.80	88.00	97.00	89.38	9.20	4.00	0.876	0.987	88.00			
20 s	depr	92.33	93.00	97.67	92.66	7.67	3.00	0.909	0.991	93.00	89.50	0.87	0.989
	kontrol	87.33	89.00	95.67	88.16	12.67	3.67	0.861	0.985	89.00			
	panik	93.33	90.67	98.00	91.98	6.67	3.33	0.903	0.987	90.67			
	ptsd	87.67	85.00	96.67	86.31	12.33	5.00	0.849	0.983	85.00			

**Table 8:** AlexNet + SVM Multi-class Classification Performance Across Different Segment Lengths.

Segment Length	Class	Prec. (%)	Recall (%)	Spec. (%)	F1 (%)	FDR (%)	FOR (%)	MCC	AUC	Class Acc. (%)	Overall Acc. (%)	Overall MCC	Micro-AUC
5 s	depr	98.25	98.41	99.40	98.33	1.75	0.54	0.978	1.00	98.41	**97.26**	**0.96**	**1.00**
	kontrol	96.56	96.00	98.84	96.28	3.44	1.35	0.950	1.00	96.00			
	panik	98.32	97.71	99.47	98.02	1.68	0.72	0.974	1.00	97.71			
	ptsd	95.95	96.91	98.61	96.43	4.04	1.05	0.952	1.00	96.91			
10 s	depr	96.48	96.16	98.74	96.32	3.51	1.37	0.950	1.00	96.16	95.06	0.93	0.99
	kontrol	93.18	95.66	97.48	94.40	6.82	1.57	0.924	0.99	95.66			
	panik	95.08	95.28	98.72	95.18	4.91	1.77	0.927	0.99	95.28			
	ptsd	95.55	93.16	98.44	94.34	6.05	2.44	0.913	0.99	93.16			
15 s	depr	94.54	95.25	98.13	94.89	5.46	1.61	0.932	0.99	95.25	93.16	0.91	0.99
	kontrol	90.14	93.75	96.52	91.89	9.86	2.15	0.891	0.99	93.75			
	panik	95.18	93.68	98.50	94.43	4.81	1.99	0.927	0.99	93.68			
	ptsd	93.02	90.00	97.71	91.48	6.98	3.35	0.887	0.99	90.00			
20 s	depr	92.73	93.66	97.51	93.20	7.26	2.15	0.909	0.99	93.66	90.97	0.88	0.98
	kontrol	87.02	91.66	95.36	89.28	12.98	2.88	0.856	0.98	91.66			
	panik	93.59	92.28	98.00	92.93	6.41	2.43	0.907	0.98	92.28			
	ptsd	90.87	86.33	97.06	88.54	9.12	4.56	0.849	0.98	86.33			

**Table 9:** GoogleNet + SVM Multi-class Classification Performance Across Different Segment Lengths.

Segment Length	Class	Prec. (%)	Recall (%)	Spec. (%)	F1 (%)	FDR (%)	FOR (%)	MCC	AUC	Class Acc. (%)	Overall Acc. (%)	Overall MCC	Micro-AUC
5 s	depr	98.41	98.41	99.46	98.41	1.58	0.54	0.979	1.00	98.41	**96.35**	**0.95**	**0.99**
	kontrol	94.56	94.25	98.16	94.48	5.43	1.95	0.925	0.99	94.25			
	panik	98.32	97.98	99.47	98.15	1.67	0.64	0.976	1.00	97.98			
	ptsd	94.20	94.83	98.02	94.51	5.79	1.76	0.927	0.99	94.83			
10 s	depr	94.56	95.66	98.13	95.11	5.44	1.47	0.935	0.99	95.66	93.71	0.92	0.99
	kontrol	93.26	92.33	97.74	92.79	6.73	2.59	0.904	0.99	92.33			
	panik	95.19	93.86	98.50	94.95	4.80	1.94	0.928	0.99	93.33			
	ptsd	91.92	93.00	97.23	92.46	8.07	2.38	0.899	0.99	93.00			
15 s	depr	92.28	92.75	97.37	92.51	7.71	2.46	0.900	0.99	92.75	91.33	0.88	0.98
	kontrol	88.38	93.25	95.84	90.75	11.61	2.33	0.876	0.98	93.25			
	panik	91.82	91.57	97.41	91.70	8.18	2.66	0.891	0.98	91.57			
	ptsd	93.10	87.75	97.79	90.34	6.90	4.07	0.873	0.98	87.75			
20 s	depr	91.00	91.00	96.94	91.00	10.23	3.05	0.867	0.99	91.00	90.31	0.86	0.98
	kontrol	89.93	89.33	96.61	89.63	10.07	3.76	0.860	0.98	89.33			
	panik	89.89	90.52	96.71	90.21	10.11	3.13	0.870	0.98	90.52			
	ptsd	89.66	89.66	96.49	89.66	10.33	3.65	0.860	0.98	89.66			

**Table 10:** ResNet50 + SVM Multi-class Classification Performance Across Different Segment Lengths.

Segment Length	Class	Prec. (%)	Recall (%)	Spec. (%)	F1 (%)	FDR (%)	FOR (%)	MCC	AUC	Class Acc. (%)	Overall Acc. (%)	Overall MCC	Micro-AUC
5 s	depr	98.74	98.25	99.58	98.50	1.26	0.60	0.980	1.00	98.25	**97.05**	**0.97**	**1.00**
	kontrol	95.28	95.92	98.39	95.60	4.72	0.43	0.960	1.00	95.91			
	panik	98.25	98.60	99.44	98.42	1.66	0.45	0.980	1.00	98.59			
	ptsd	95.98	95.50	98.64	95.74	1.29	1.52	0.961	1.00	95.50			
10 s	depr	95.72	96.83	98.53	96.27	4.28	1.08	0.950	1.00	96.83	95.24	0.94	0.99
	kontrol	94.05	94.83	97.97	94.44	5.95	1.76	0.926	0.99	94.83			
	panik	96.78	94.91	99.00	95.84	3.22	1.60	0.945	0.99	94.91			
	ptsd	94.66	94.50	98.19	94.58	5.34	1.86	0.927	0.99	94.50			
15 s	depr	96.00	96.00	98.64	96.00	4.00	1.36	0.946	1.00	96.00	93.29	0.91	0.99
	kontrol	89.81	92.50	96.44	91.13	10.19	2.57	0.881	0.99	92.50			
	panik	96.00	94.74	98.75	95.36	4.00	1.66	0.939	0.99	94.73			
	ptsd	91.60	90.00	97.20	90.79	8.40	3.37	0.877	0.99	90.00			
20 s	depr	94.04	94.67	97.97	94.35	5.96	1.81	0.924	0.99	94.66	91.48	0.89	0.99
	kontrol	89.04	89.33	96.27	89.19	10.96	3.62	0.855	0.98	92.00			
	panik	94.35	93.68	98.22	94.01	5.65	2.00	0.921	0.99	93.68			
	ptsd	88.63	88.33	96.16	88.48	11.37	3.95	0.846	0.98	88.33			

**Table 11:** Statistical Significance Analysis of Model Comparisons.

**Impact of SVM Integration**				
**Comparison**	**Δ Acc (%)**	***t*****-Test** ***p***	**Wilcoxon** ***p***	**Significant**
ResNet50 + SVM vs. ResNet50	+0.49	0.009 **	0.063	Yes **
GoogLeNet + SVM vs. GoogLeNet	+0.20	0.548	0.625	No
AlexNet + SVM vs. AlexNet	+2.41	0.007 **	0.063	Yes **
**Comparison of Hybrid Models**				
ResNet50 + SVM vs. GoogLeNet + SVM	+0.70	0.086	0.125	No
ResNet50 + SVM vs. AlexNet + SVM	−0.21	0.037 *	0.063	Marginal *
GoogLeNet + SVM vs. AlexNet + SVM	−0.91	0.054	0.125	No

* *p* < 0.05, ** *p* < 0.01.

**Table 12:** McNemar’s Test Results for Hybrid Model Comparisons.

Comparison	Discordant	$\chi^{2}$	*p*-Value	Winner	Δ Samples
ResNet50 + SVM vs. AlexNet + SVM	180	0.45	0.502	—	+10
ResNet50 SVM vs. GoogLeNet + SVM	207	4.95	0.026 *	ResNet50	+33
AlexNet + SVM vs. GoogLeNet + SVM	199	8.86	0.003 **	AlexNet	+43

* *p* < 0.05, ** *p* < 0.01.

**Table 13:** Error Analysis Summary for 5-s Segments.

	ResNet50 + SVM	GoogLeNet + SVM	AlexNet + SVM
Overall Accuracy	97.05%	96.35%	97.26%
Total Errors	140	173	130
Primary Confusion Patterns:			
PTSD ↔ Control	101 (72.1%)	130 (75.1%)	83 (63.8%)
Depression ↔ Panic	33 (23.6%)	37 (21.4%)	38 (29.2%)
PTSD ↔ Other Disorders	1 (0.7%)	2 (1.2%)	3 (2.3%)
Individual Error Breakdown:			
PTSD → Control	54	61	35
Control → PTSD	47	69	48
Depression → Panic	18	18	17
Panic → Depression	15	19	21
Other errors	6	6	9

Note: Percentages indicate proportion of total errors. PTSD-Control confusion dominates (64–75%) across all models. PTSD differentiation from depression and panic remains highly accurate (<3% confusion combined).

**Table 14:** Performance comparison of traditional machine learning approaches with handcrafted statistical features.

ML Model	Class	Prec. (%)	Recall (%)	Spec. (%)	F1 (%)	AUC	Class Acc. (%)	Overall Acc. (%)
SVM (Linear)	depr	43.48	50.00	77.97	46.51	0.770	50.00	**44.30**
	kontrol	43.48	50.00	77.97	46.51	0.691	50.00	
	panik	47.06	42.11	85.00	44.44	0.717	42.11	
	ptsd	43.75	35.00	84.75	38.89	0.625	35.00	
KNN (Cubic)	depr	34.38	55.00	64.41	42.31	0.609	55.00	32.91
	kontrol	41.18	35.00	83.05	37.84	0.725	35.00	
	panik	26.67	21.05	81.67	23.53	0.582	21.05	
	ptsd	26.67	20.00	81.36	22.86	0.587	20.00	
Ensemble (Bagged Tree)	depr	50.00	60.00	79.66	54.55	0.738	60.00	40.51
	kontrol	45.46	50.00	79.66	47.62	0.791	50.00	
	panik	37.50	31.58	83.33	34.29	0.623	31.58	
	ptsd	23.53	20.00	77.97	21.62	0.507	20.00	
NN (Trilayered)	depr	36.84	35.00	79.66	35.90	0.597	35.00	31.65
	kontrol	31.25	25.00	81.36	27.78	0.500	25.00	
	panik	42.11	42.11	81.67	42.11	0.661	42.11	
	ptsd	20.00	25.00	66.10	22.22	0.405	25.00	
Linear Discriminant	depr	44.44	40.00	83.05	42.11	0.735	40.00	34.18
	kontrol	16.67	10.00	83.05	12.50	0.459	10.00	
	panik	42.31	57.90	75.00	48.89	0.718	57.90	
	ptsd	26.09	30.00	71.19	27.91	0.500	30.00	
Naive Bayes (Gaussian)	depr	46.15	30.00	88.14	36.36	0.725	30.00	37.97
	kontrol	38.46	25.00	86.44	30.30	0.541	25.00	
	panik	34.48	52.63	68.33	41.67	0.675	52.63	
	ptsd	37.50	45.00	74.58	40.91	0.594	45.00	

## Data Availability

The dataset analyzed in this study is unavailable for public release owing to ethical and privacy constraints.

## Abbreviations

The following abbreviations are used in this manuscript:

ECG	Electrocardiogram
CWT	Continuous Wavelet Transform
CNN	Convolutional Neural Network
SGD	Stochastic Gradient Descent
SGDM	Stochastic Gradient Descent with Momentum

PCA	Principal Component Analysis
MCC	Matthews Correlation Coefficient
AUC	Area Under the Curve
FDR	False Discovery Rate
FOR	False Omission Rate
PTSD	Post-Traumatic Stress Disorder
Hz	Hertz
FC	Fully Connected
